# Dental Empiricism, Past and Present

**Published:** 1861-07

**Authors:** Felix Weiss


Dental Empiricism, Past and Present—By *Felix Weiss*, M. C. D. E.—Although it can not be denied that the dental profession has made, of late years, great progress towards its permanent establishment as one of the most useful and honorable institutions of this or any country—(a progress not only in the art itself, but in the general education of its professors)—yet it is melancholy to reflect upon the degradation and selfishness which still attach to several of its members. "Go forward," says the first President of the Cincinnati Dental Association—"Go forward in the good cause, unintimidated by the scepticism of the faithless, the fear of the timid, or the apathy of the selfish." But to make that progress secure, we must have unity; and to make that unity respected, we must endeavor so to educate our members, and the public generally, that empiricism may not only be despised, but cease to be profitable.

Some fifty years ago, all that was needed to commence the practice of a dentist was unblushingly to profess yourself one —few, indeed, were those who, qualified by study and skilled by experience, conscientiously endeavored to carry it out in their every-day practice. The profession was regarded by the many simply as a money-making business, and had the great virtue of being novel. The public, at all times ready to be gulled, fostered the empiric, flocked to his house, paying him nearly any fee he might demand,—only too willing if he would undertake their case at all. Fifty years ago, an adventurer of this stamp (I take him as an illustration of his class) visited Paris, that he might pick up a smattering of this new and profitable calling. At the end of three months, returning to London, by specious advertisements, by well-got-up circulars, he soon managed to make himself known; his establishments were multiplied, until at length he possessed several houses in London, and had travelers scouring the country for patients in every direction. It is not our office to rake up the grievances of the past, excepting so far as they may be profitable to pointing an example for the future, but the statistics afforded by these notorious empirics are worthy a moment's reflection. The ordinary charge for a set of teeth was one hundred guineas, and it is notorious that in some instances more than twice that amount was demanded ; all other charges were in proportion, a common price being five guineas a tooth. Lotions for the gums were made up and sold at one and two guineas a bottle, and boxes of powder at similar exorbitant prices.

Now, if in return for these enormous fees the work had been really well executed, some excuse might have been pleaded, as the science was young and its professors greedy ; but, to a certain extent, any rubbish was put into the mouth—badly-fitted plates of gold and silver, marvelously stinted in quantity and disgracefully low in quality, with ill-adapted blocks of bone, and sometimes natural, but more frequently carved teeth. The object of the empirics was to see a patient but once, and make as much as possible out of the first transaction. The class of patients that visited them were for the most part rich, and moved in the best society ; indeed, some of the highest members of the nobility. The stratagems resorted to by these harpies would alone fill a volume. Carriages were hired to be continually driving up to their doors, and there to remain, two or three at a time. Well-dressed visitors were paid to fill their waiting-rooms, and to expatiate upon their skill ; entertainments of all kinds were given, while their well-worded books and gilt-edged circulars found their way into every house, and their advertisements into every paper.

Can it be wondered at that, with all these auxiliaries at work, the receipts netted by some amounted to as much as £30,000 a year ? Can it cause any surprise that the name of dentist was associated with wealth? But it will be equally apparent that such proceedings were gradually bringing the whole body into disgrace ; distrust was creeping over the public mind, the word dentist became a questionable title, and even twenty years afterwards, when many able men commenced practice, they were hardly received with anything like the respect attached to other professions.

Having now glanced at the dental empirics of the past, let us for a moment examine those in practice at the present day; and we fear that while doing so we shall be compelled, with sorrow, to admit that their character remains unaltered. Is it not notorious that monied men are entering the profession, employing workmen and assistants, and setting aside large sums for the purposes of advertisement, etc., who possess not a scintilla of professional knowledge; and that the public patronise them as of old, pay them heavy fees, robbing in the greater number of instances the honest practitioner, and injuring the public mind? But, after all, the question naturally arises, How is this to be avoided? Educate the public, and let every enlightened member of the profession in his private capacity take every opportunity of impressing upon society the necessity of a charter, with its pains and penalties, and endeavor to inculcate a thorough distrust in advertised ability.

Let us now inquire what constitutes dental empiricism; and we shall try to prove that all systems of obtaining practice, excepting through recommendation, is alike disreputable and dangerous to society. Advertisements may be divided into two classes—inoffensive and offensive. The inoffensive advertisement is simply a change of address, or a card. The offensive is full of professions for the most part untrue, and assertions intended simply to attract attention;—such as the exclusive use of patents where no patents exist; the possession of valuable preparations, it being well known that the materials used in dentistry are alike open to all; methods of construction under a variety of names, assumed to be only in the possession of the advertiser. We have likewise a series of stereotyped phrases, so grossly untrue that no one possessing one spark of integrity would allow their names to be attached to them; the phrases being not only opposed to scientific experience, but even repugnant to common sense.

The advertiser depends upon his advertisements; and herein lies the grand distinction between him and the honest practitioner. The empiric never desires to see his patient a second time; the advertisement that brought the dupe of to-day will bring him another to-morrow. The non-advertiser feels that his reputation—indeed, his very existence—depends upon his acting honorably to his patient. He can only increase his connection by the recommendation of one to another. The patient may feel confidence that he is dealing with a man who lives only by his good name ; not so the advertiser, however honorable he may be (and we will allow they are not all dishonorable),—he obtains his patients in so questionable a manner, that his skill must at all times remain doubtful. If he is a clever man, what need has he of advertisement at all? Ability will sooner or later meet with some amount of recognition ; therefore the dentist who requires advertisement must necessarily be the inferior practitioner, executing the worst work, relying upon his advertisement, without which he would be without patients, as he is without skill.

The answer to much that we have advanced will be, that the private and high-priced dentist (as the empirics will term him) demands higher fees, and complains at the charges made by the advertiser. This we unhesitatingly dispute, and freely declare that the charges, on the whole, made by the private practitioner are, if anything, less than those of the empiric. It is true, he will not condescend to operate after the advertiser's fashion. Stopping a tooth is not merely filling it with any description of paste ; and artificial teeth should possess some amount of finish,—the cheapest not being always considered by rational beings the best. Again, the advertised prices bear no proportion to the prices really charged, excepting in a few solitary instances; for if a man can afford to spend from £200 to £2,000 a year in advertisements, his profits must be obtained from some one.

The organization of a Dental College and the formation of a Dental Society are not calculated to check the empiric, excepting in as far as they educate the general public. A vast number of young practitioners have been accustomed to send out a few circulars ; but upon joining these bodies they have discontinued doing so, advertising being opposed to the laws they have subscribed to ; so that the empiric has even a larger sphere of operation than he had before the formation of these societies. And this is one of the grand reasons why we should pray for a code of pains and penalties. Men who are honorably endeavoring to make a position, feel acutely the injustice done to them by the public, and, but too frequently, seeing the success that attends the advertising charlatans (no matter how inferior their ability), are led to join them. Upon these arguments we found our conviction that dental advertisements are injurious to the public, and ought in every way to be discountenanced.

Another mode of gaining notoriety, or, more correctly speaking, of attracting attention, is by the show-case, which we must also unhesitatingly condemn, and upon these grounds; —The show-case does not necessarily contain specimens of work executed by its exhibitor. Every practical dentist is aware that these cases may be obtained ready fitted up with work in all styles. But even granting that it is the work it professes to be, it does not follow, however well it may be executed, that such work will fit the mouth, or could be worn with comfort. Some may argue that the show-case is no more than the shop-window, in which is exhibited the wares of the artificer. But the cases are widely different. We may see a chair or a table, a piece of jewellery or a garment, exhibited for sale; we can purchase the chair and place it in our rooms. But such is not the case with a set of teeth. Every customer must be separately fitted, and in no instance could the dentist remove from his show-case a piece of work and transfer it to the mouth of a strange patient. We know that many regard the show-case as simply emblematic of the dentist's calling; but upon these grounds we should excuse the exhibition of a golden tooth or a gigantic pair of forceps.

We have penned these few lines in no malignant spirit, but have endeavored to bring forward undeniable arguments in support of our views. To the younger members of the profession, those fighting (perhaps despondingly) the battle of life, we would remark that the advertising empiric's successes are not now what they used to be; every day, every hour, the public are becoming better informed, and we sincerely believe the day is not far distant when the empiric will cease altogether to be patronised. Let the young practitioner, though he possess but half-a-dozen patients, do all in his power to convince them that he is thoroughly conscientious; let him also take every opportunity of increasing his store of knowledge; and in the end he will have little or no cause to complain, though his neighbor disreputably canvasses his position inch by inch. Many have tried by advertisement to obtain more rapidly a connection, but have since returned to the true walk of a respectable practitioner, bitterly lamenting their folly.

To those already advertising, we would inquire whether it were not better for them to cease to do so before it is too late; to gather together what connection they possess, and save themselves the thousands they expend, while lowering themselves in the eyes of their professional brethren.

In conclusion, to all we would express our conviction that by unity, energy and perseverance, the public mind will become so enlightened, that dental empiricism can no longer exist, and that every one endeavoring to carry out this good work will deserve not only the thanks of the profession at large, but of every right-minded member of society.—*Dental Review.*

